# Research misconduct in health and life sciences research: A systematic review of retracted literature from Brazilian institutions

**DOI:** 10.1371/journal.pone.0214272

**Published:** 2019-04-15

**Authors:** Rafaelly Stavale, Graziani Izidoro Ferreira, João Antônio Martins Galvão, Fábio Zicker, Maria Rita Carvalho Garbi Novaes, César Messias de Oliveira, Dirce Guilhem

**Affiliations:** 1 Department of Nursing, College of Health Sciences, University of Brasilia, Brasília, Federal District, Brazil; 2 Department of Statistics, Telecomunicações do Brasil – Telebrás, Brasília, Federal District, Brazil; 3 Center for Technological Development in Health, Oswaldo Cruz Foundation, Brasília, Federal District, Brazil; 4 Department of Nursing, College of Health Sciences, Health Sciences Education and Research Foundation – ESCS/Fepecs, Brasília, Federal District, Brazil; 5 Department of Epidemiology & Public Health, Institute of Epidemiology & Health Care, University College London, London, United Kingdom; University of Oxford, UNITED KINGDOM

## Abstract

**Background:**

Measures to ensure research integrity have been widely discussed due to the social, economic and scientific impact of research integrity. In the past few years, financial support for health research in emerging countries has steadily increased, resulting in a growing number of scientific publications. These achievements, however, have been accompanied by a rise in retracted publications followed by concerns about the quality and reliability of such publications.

**Objective:**

This systematic review aimed to investigate the profile of medical and life sciences research retractions from authors affiliated with Brazilian academic institutions. The chronological trend between publication and retraction date, reasons for the retraction, citation of the article after the retraction, study design, and the number of retracted publications by author and affiliation were assessed. Additionally, the quality, availability and accessibility of data regarding retracted papers from the publishers are described.

**Methods:**

Two independent reviewers searched for articles that had been retracted since 2004 via PubMed, Web of Science, Biblioteca Virtual em Saúde (BVS) and Google Scholar databases. Indexed keywords from Medical Subject Headings (MeSH) and Descritores em Ciências da Saúde (DeCS) in Portuguese, English or Spanish were used. Data were also collected from the Retraction Watch website (www.retractionwatch.com). This study was registered with the PROSPERO systematic review database (CRD42017071647).

**Results:**

A final sample of 65 articles was retrieved from 55 different journals with reported impact factors ranging from 0 to 32.86, with a median value of 4.40 and a mean of 4.69. The types of documents found were erratum (1), retracted articles (3), retracted articles with a retraction notice (5), retraction notices with erratum (3), and retraction notices (45). The assessment of the Retraction Watch website added 8 articles that were not identified by the search strategy using the bibliographic databases. The retracted publications covered a wide range of study designs. Experimental studies (40) and literature reviews (15) accounted for 84.6% of the retracted articles. Within the field of health and life sciences, medical science was the field with the largest number of retractions (34), followed by biological sciences (17). Some articles were retracted for at least two distinct reasons (13). Among the retrieved articles, plagiarism was the main reason for retraction (60%). Missing data were found in 57% of the retraction notices, which was a limitation to this review. In addition, 63% of the articles were cited after their retraction.

**Conclusion:**

Publications are not retracted solely for research misconduct but also for honest error. Nevertheless, considering authors affiliated with Brazilian institutions, this review concluded that most of the retracted health and life sciences publications were retracted due to research misconduct. Because the number of publications is the most valued indicator of scientific productivity for funding and career progression purposes, a systematic effort from the national research councils, funding agencies, universities and scientific journals is needed to avoid an escalating trend of research misconduct. More investigations are needed to comprehend the underlying factors of research misconduct and its increasing manifestation.

## Introduction

Research integrity relies on rigorous methodological approaches during planning, conduct, documentation and reporting of studies [1]. Practices known to harm these steps are classified as research misconduct [2], [3]. It has become more common for studies addressing the impact of misconduct to be published as a warning to the scientific community [4], [5], [6]. In 2012, Fang and colleagues conducted a systematic review of retracted publications in the field of biomedical and life sciences using PubMed. Their findings showed that most of the retractions were due to fraud, and they addressed the impact of these findings since these studies are mainly publicly funded [4].

Research misconduct occurs when plagiarism, data manipulation, fabrication, poor study reporting, and lack of transparency are part of the scientific process [2]. These acts have been found to compromise the validity and reliability of research results [7], [8], [9]. On many occasions, these faults have led to a retraction notice. The publication of retraction notices intends to alert readers to serious errors—unintentional or of misconduct nature—that result in unreliable conclusions [7]. The purpose of retraction notices is also to avoid the use of these studies as a basis for future investigations, except for research about scientific integrity itself. Additionally, retractions are an important tool to evaluate scientific production, and the study of retractions supports measures to avoid error and misconduct.

Misconduct has scientific, social and economic impacts [5], [8], [10]. Economically, it has been estimated that billions of dollars have been wasted on funding studies based on retracted publications [11]. Socially, it affects evidence-based medicine by exposing study volunteers and the population as a whole to wrong medical decisions [10]. Scientifically, further investigations based on unreliable findings and unethical research leads to untrustworthy conclusions, compromising the advances of scientific knowledge [9], [12], [13]. Therefore, corrupted research conducts may generate a chain of misconduct [6], [10].

Financial support for health and life sciences research has steadily increased in Brazil, which has been followed by a rising number of scientific publications. Simultaneously, there have been a growing number of retracted publications, raising concerns about the quality and reliability of these articles. The first retraction reported in health and life sciences from Brazilian institutions was a paper about nursing that was published in 2004 [14]. At the time, the author admitted to plagiarism. Since then, other cases of research misconduct have been discovered, generating apprehension about the scientific advances in the country.

Brazil is a member of the BRICS (Brazil, Russia, India, China, South Africa) cooperative group that is responsible for some of the 1% most cited publications in the world [15]. Although the citation impact of the country is below the global average, it increased 15% in the past six years [15]. The publications with higher impact ratings were performed mainly in collaboration with other institutions from the BRICS. The scientific influence of the country, as well as its participation in collaboration funds and networks for promoting health research, is growing worldwide [15].

The understanding of research integrity and research misconduct varies institutionally and culturally [16], [17], [18], so it is important to understand the factors underlying the retractions of Brazilian scientific publications and the notable increase in retractions.

Despite the relevance of research misconduct and the awareness of breaches of research integrity, the analysis of retracted publications in Brazil is quite new. In this context, this systematic review proposed the following research question: What are the main reasons for retracted publications in the field of health and life sciences that were published by researchers who are affiliated with Brazilian institutions? Answering this research question will pave the way for future investigations about research integrity in Brazil by recognizing the particularities of the country.

This review intended to characterize the underlying causes of retraction, to assess the extent of research misconduct, to support discussions of possible solutions, and ultimately, to promote further investigations. To carry out this review, data were collected regarding reasons for retraction, temporal trends from publication to retraction, citation pattern after retraction, and the impact factors and ethical guidelines endorsements of the journals. Additionally, this review evaluated the quality of retraction notices considering whether complete information was provided in accordance with the COPE guidelines [1]–a fundamental aspect of research transparency.

## Materials and methods

### Protocol and registration

This review protocol was registered with PROSPERO (CRD42017071647).

### Information source

The screening of eligible publications was performed from late July to early August 2017 in accordance with the preapproved registered protocol.

### Search strategy

Details of the search strategy are available via the following link: https://www.crd.york.ac.uk/PROSPEROFILES/71647_STRATEGY_20170610.pdf.

### Study selection

For this review, retraction notices that were published from January 2004 until August 2017 regarding articles that had at least one author that was affiliated with a Brazilian institution, irrespectively to their authorship position and regardless of the publication year of the original article, were selected. The start date was the publication year of the first retracted article in nursing science that was written by authors affiliated with a Brazilian institution [14].

Studies in the field of life and health sciences following the *Brazilian National Council for Scientific and Technological Development*, CNPq (from the Portuguese, Conselho Nacional de Desenvolvimento Científico e Tecnológico), classification [19] that were published in English, Portuguese or Spanish in national or international journals were eligible for this review.

Despite their study design, all retracted articles, with complete or incomplete retraction notice information according to the Committee of Publication Ethics (COPE) guidelines [2], were eligible for this review when they were in accordance with the protocol. Retraction notices, articles with a retraction notice attached or any sort of information indicating a retraction were considered for data collection. Studies regarding research integrity were excluded, as well as studies related to other fields of scientific knowledge.

### Sampling and data collection process

Two independent reviewers searched for retracted articles via the PubMed, Web of Science and Brazilian Virtual Library of Health (BVS) databases. Google Scholar and the Retraction Watch [20] website were searched to identify additional publications and gray literature. The last database is an open access portal reporting retracted papers worldwide. The results were compared, and a consolidated list of retracted articles was produced according to the protocol.

Data were collected and analyzed according to reason for retraction, time trend from publication to retraction, citation pattern after retraction, journal impact factor, quality of retraction notice information, author’s affiliation and adherence to either COPE or CONSORT guidelines on ethics and standard reporting.

### Data collection rationale

1. **Publication year and retraction year trend**: The time between the date of publication and the date of retraction was calculated in years. Articles published and retracted in the same year were considered to have a time of 0. Publications without complete information regarding these dates were labeled as “not applicable” for this analysis.
2. **Author’s affiliation**: This analysis was limited to one author per paper. Data were collected from the last authors because they are typically responsible for mentoring and supervising the research planning, conduct and reporting [21]. Three articles were excluded from this analysis because the last author was not affiliated with a Brazilian institution.
3. **Journal’s name and impact factor (IF)**: The impact factor over the last 5 years was collected from Thompson and Reuters’s indicators. Previous research has shown an increase in the citation of retracted papers when they were published in high impact journals [9]. This review investigated whether the same pattern exists in Brazilian publications.
4. **Ethical and reporting guidelines endorsement**: It was assumed that journals endorsed by either CONSORT or COPE guidelines followed ethical guidelines.
5. **Area of study**: The health and life sciences were categorized into the following sub groups: medical science, biological science, nutrition, dentistry, sports science, nursing science, physiotherapy, and pharmacology.
6. **Retraction indicator**: The presentation of retraction notices or retracted articles reflected how editors and databases did or did not facilitate their visibility. Transparency was ensured when retraction notices were attached to the original article and had a clear warning of retraction/withdrawal.
7. **Reasons for retraction**: The reasons for retraction were classified as a) error (inappropriate study design, data collection or report); b) fraud (data or image manipulation); c) author’s dispute (publications without the consent or recognition of all authors, sponsors or industry manufacturers of the tested product); d) duplicated publication (when authors or editors published the same article more than once); e) irregular citation pattern or citation staking (artifice used to upgrade the impact factor of a journal); f) unknown (reason for retraction was not mentioned); g) plagiarism (image, text or unspecified forms of plagiarism) and; h) no informed consent was obtained for the use and publication of images of participants.
8. **Retracted by**: Retraction notices are expected to acknowledge who retracted the article. Retractions by authors indicate good faith and are considered as retractions due to an honest mistake. Retractions by editors, depending on the reason, may indicate honest mistakes from the editorial board or misconduct from the authors.
9. **Retraction endorsement by authors**: Authors usually participate and/or agree with the wording of the retraction. Report of participation of authors and their endorsement indicates transparency of the retraction process.
10. **Citation pattern of retracted articles**: The number of times an article has been cited reflects its visibility and possible impact on the scientific community [22]. Therefore, the citation pattern before and after retraction was analyzed by calculating the mean citations per year from the date of publication to the date of retraction for each article. Similarly, the mean citations per year from the date of retraction to 2017 were also calculated. For comparison purposes, articles with a higher mean number of citation per year before retraction were considered to have a *positive-citation pattern*, while those with a higher mean number of citations per year after retraction were considered to have a *negative-citation pattern*.
11. **Quality of retraction notices**: According to the COPE recommendations [2], [7], retraction notices must contain: the date of retraction, motives for the retraction, whether the retraction was endorsed by the authors, who requested the retraction, and the proper citation of the original article in the retraction notice. A complete report of this information accounts for a high-quality retraction notice.

### Report

The PRISMA statement checklist was used to assure the quality of this systematic review. The checklist is provided as S1 Table. Some topics did not apply for this study considering that this review evaluated only retraction notices and excluded the original articles. Consequently, the methods used to assess the risk of bias of the individual studies, summary measures, synthesis of results and risk of bias across studies was not used.

### Statistical analysis

The Shapiro-Wilk normality test was conducted for the citation pattern before and after retraction and the correlation between the citation pattern and the impact factor of the journals. These variables exhibited a non normal distribution. Hence, the Spearman correlation test and a descriptive analysis were performed using the R statistical program version 3.4.2 and Excel for Mac 2011, version 14.4.3. S1 File of the conducted tests is available.

## Results

### Retraction notice selection

**Fig 1:**
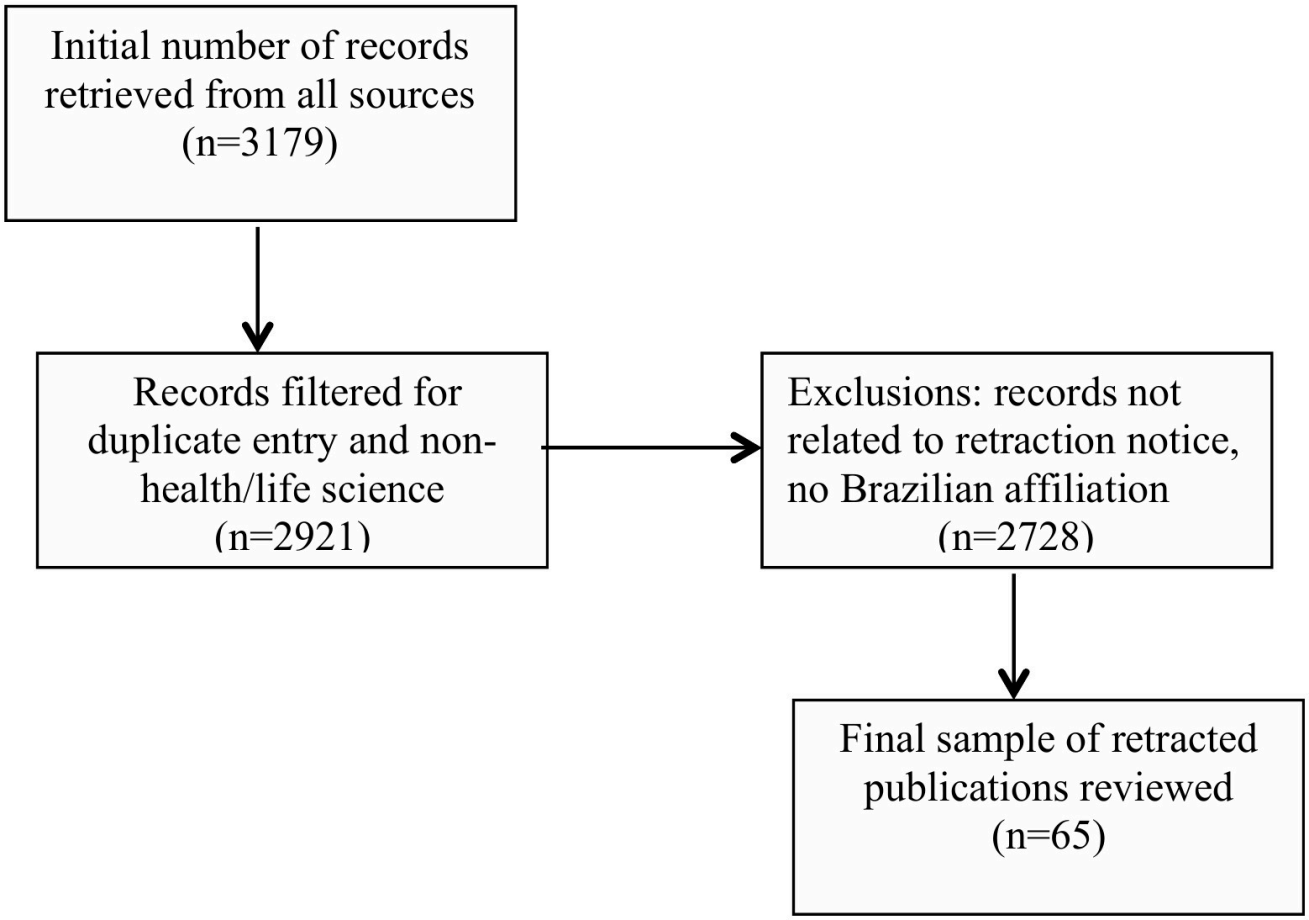
Flowchart of study identification and eligibility of retracted articles. Study selection flowchart showing initial number of records to final sample retrieved for analysis.

A final sample of 65 retracted articles was retrieved (Fig 1) from 55 different journals with an impact factor range of 0–32.86 and a mean impact factor of 4.7. The types of documents that were included were erratum (n = 1), retracted article (n = 3), retracted article with its retraction notice attached (n = 5), retraction notice with erratum (n = 3) and retraction notice (n = 45). The search using the Retraction Watch Blog [13] added 8 articles that were not identified by the search strategy using the bibliographic databases.

The retracted publications covered a wide range of studies. Experimental studies (n = 40) and literature reviews (n = 15) accounted for 84.6% of the included articles Table 1. Studies conducted in the field of medical science accounted for 52% of the retrieved articles. Medical science was the field with the largest number of retractions (n = 34) followed by biological sciences at 26% (n = 17), dentistry 7.7% (n = 5), sports sciences at 3% (n = 2), pharmacology at 3% (n = 2), nutrition at 1.5% (n = 1), nursing sciences at 1.5% (n = 1), and physiotherapy at 1.5% (n = 1).

**Table 1:** Type of study according to area of study.

Study type/area	N (%)
**Experimental**	40 (61.5%)
Medical Sciences	19
Biological Sciences	14
Dentistry	3
Sports Sciences	2
Physiotherapy	1
Nutrition	1
**Literature review**	15 (23.0%)
Medical Sciences	12
Pharmacology Sciences	2
Biological Sciences	1
**Observational**	6 (9.2%)
Biological Sciences	2
Medical Sciences	2
Nursing Sciences	1
Dentistry	1
**Case study**	2 (3.0%)
Nutrition	1
Dentistry	1
**Meta-analysis**	1 (1.5%)
Medical Sciences	1
**Systematic review**	1 (1.5%)
Medical Sciences	1
**Total**	**65**

#### Ethical and standard reporting guidelines

Out of the 65 journals with published retracted notices, only 7 clearly complied with the COPE and CONSORT guidelines. A total of 41.5% of the selected journals were not member of COPE or part of CONSORT’s list. Still, reference to these two main ethical and reporting guidelines recommendations was found in the *Guide for Authors* section of these journals.

#### Affiliation, number of retractions and area of study of the authors

A total of 26 Brazilian institutions had at least one research article retracted. Of these institutions, 20 (77%) were public institutions, 5 (19%) were private institutions and 1 (4%) was a nonprofit organization. The University of São Paulo was the institution with the highest number of retracted publications (n = 17), followed by the University of Campinas (n = 16). Both are leading Brazilian academic institutions with the highest scientific productivity [15]. Of the 62 articles analyzed, 48 (77.4%) were published by authors affiliated with institutions located in southeastern Brazil. The University of Campinas (São Paulo) also accounted for the highest number of retractions by author Table 2. The largest number of postgraduate programs in the country is concentrated in the southeastern region of Brazil [23]. One author had 8 retractions during the studied period. Plagiarism was the main cause for retractions related to the two authors with most retractions that were affiliated with this university Table 3.

**Table 2:** Distribution of retracted publications by affiliation, author and area of study^1^.

Brazilian Institution	Retracted Publication N (%)^2^	Last Author	Number of Retracted Publications N
**Universidade de São Paulo (USP)**	17 (27.4%)	Gomes A	4
		Curi R	2
		Rocha e Silva M	2
		Santo D. S.	1
		Soares AM	1
		Marchini JS	1
		Pereira LV	1
		Oliveira MN.	1
		Miguel EC.	1
		SVerjovski-Almeida S	1
		Zuben CJV	1
		Mendonca MR	1
**Universidade Estadual de Campinas (UNICAMP)**	16 (25.8%)	Saad MJA	8
		Velloso LA	3
		Carvalheira JBC	3
		Reis SF	1
		Franchini KG	1
**Hospital Universitário Pedro Ernesto, Universidade Estadual do Rio de Janeiro (HUPE-UERJ)**	2 (3.2%)	Gomes MB	2
**Escola Bahiana de Medicina e Saúde Pública (BAHIANA)**	2 (3.2%)	Ladeia AM	1
		Pazos RMA	1
**Universidade Federal do Rio de Janeiro (UFRJ)**	2 (3.2%)	Farias MLF de.	2
**Universidade Federal do Triângulo Mineiro (UFTM)**	2 (3.2%)	Etchebehere RM	1
		Patrizzi LJ	1
**Universidade Federal do Paraná (UFPR)**	2 (3.2%)	Reichembach MT	1
		Antoniuk SA	1
**Faculdade de Ciências Médicas da Santa Casa de São Paulo**	1 (1.6%)	Alli LAC	1
**Faculdade de Medicina de Marilia (FAMEMA)**	1 (1.6%)	Stefano EJ	1
**Heart Institute (INCOR)**	1 (1.6%)	Hajjar LA	1
**Hospital do Servidor Público Estadual de São Paulo (IAMSPE)**	1 (1.6%)	Rotta JM	1
**Hospital Israelita Albert Einstein**	1 (1.6%)	Gamarra LF	1
**Project “Avulsos Malacológicos—AM”**	1 (1.6%)	Agudo-Padrón AI	1
**Centro Universitário de Várzea Grande (UNIVAG)**	1 (1.6%)	Ravagnani FCP	1
**Universidade Federal da Fronteira Sul (UFFS)**	1 (1.6%)	Mossi AJ	1
**Universidade Federal de Pernambuco (UFPE)**	1 (1.6%)	Rolim Neto, P.J.I	1
**Universidade Federal de Viçosa (UFV)**	1 (1.6%)	Silva VE	1
**Universidade Federal do Maranhão (UFMA)**	1 (1.6%)	Oliveira AE	1
**Universidade Federal Rural do SemiÁrido (UFERSA)**	1 (1.6%)	Costa LLM	1
**Universidade Paulista de Goiania (UNIP-Goiania)**	1 (1.6%)	Botelho TL	1
**Universidade Federal de Campina Grande (UFCG)**	1 (1.6%)	Campos JHBC	1
**Universidade do Vale do Itajaí (UNIVALE)**	1 (1.6%)	Menezes JT	1
**Universidade Estadual Paulista "Júlio de Mesquita Filho" (UNESP)**	1 (1.6%)	Valenti VE	1
**Universidade Federal da Bahia (UFBA)**	1 (1.6%)	Portela RW	1
**Universidade Estadual do Norte Fluminense (UENF)**	1 (1.6%)	Gomes VM	1
**Universidade de Brasília (UnB)**	1 (1.6%)	Teixeira ARL	1
**Total**	**62 (100%)**	-	**62**

^1^Specifically for this analysis, three articles were excluded because the last author was not affiliated with a Brazilian institution. These articles were considered for other analyses because they included authors who were affiliated with Brazilian institutions.

^2^ The percentage presented considerate the sixty-two articles included for this analysis.

**Table 3:** Bibliographical references and reasons for retraction of the articles by the authors with the most retractions.

Author/institution	DOI	Journal	IF	Reason for retraction
**Saad MJA** **UNICAMP**	10.2337/diab.46.12.1950	Diabetes	8.512	Image Plagiarism
	10.2337/db06-1595	Diabetes	8.512	Image Plagiarism/ Fraud
	10.1590/S0004-27302009000200004	Arquivos Brasileiros de Endocrinologia e Metabologia	1.045	Plagiarism
	10.2337/db09-1907	Diabetes	8.512	Image Plagiarism
	10.1186/s13054-016-1453-8	Critical Care	5.406	Image Plagiarism
	10.1371/journal.pbio.1002479	PloS Biology	10.7	Image Plagiarism
	10.2337/db17-rt03a	Diabetes	8.512	Image Plagiarism
	10.1371/journal.pone.0159283	PloS One	3.535	Plagiarism
**Gomes A** **USP**	10.6061/clinics/2013(10)17	Clinics	1.444	Error
	10.1590/S1516-31802012000500009	São Paulo Medical Journal	0.893	Error
	2012;20(6):367–71	Acta Ortopédica Brasileira	0.384	Citation Stacking
	10.1590/S1807-59322011001100020	Clinics	1.444	Error
**Velloso LA** **UNICAMP**	10.1074/jbc.A110.173021	Journal of Biological Chemistry	4.403	Image Plagiarism
	10.1074/jbc.A111.315218	Journal of Biological Chemistry	4.403	Image Plagiarism
	10.1074/jbc.A109.030874	Journal of Biological Chemistry	4.403	Image Plagiarism
**Carvalheira JB** **UNICAMP**	10.2337/db05-1622	Diabetes	8.512	Image Plagiarism
	10.2337/db17-rt03b	Diabetes	8.512	Image Plagiarism/ Fraud
	10.1053/j.gastro.2012.05.045	Gastroenterology	16.825	Image Plagiarism

#### Time trend between publication and retraction

**Fig 2:**
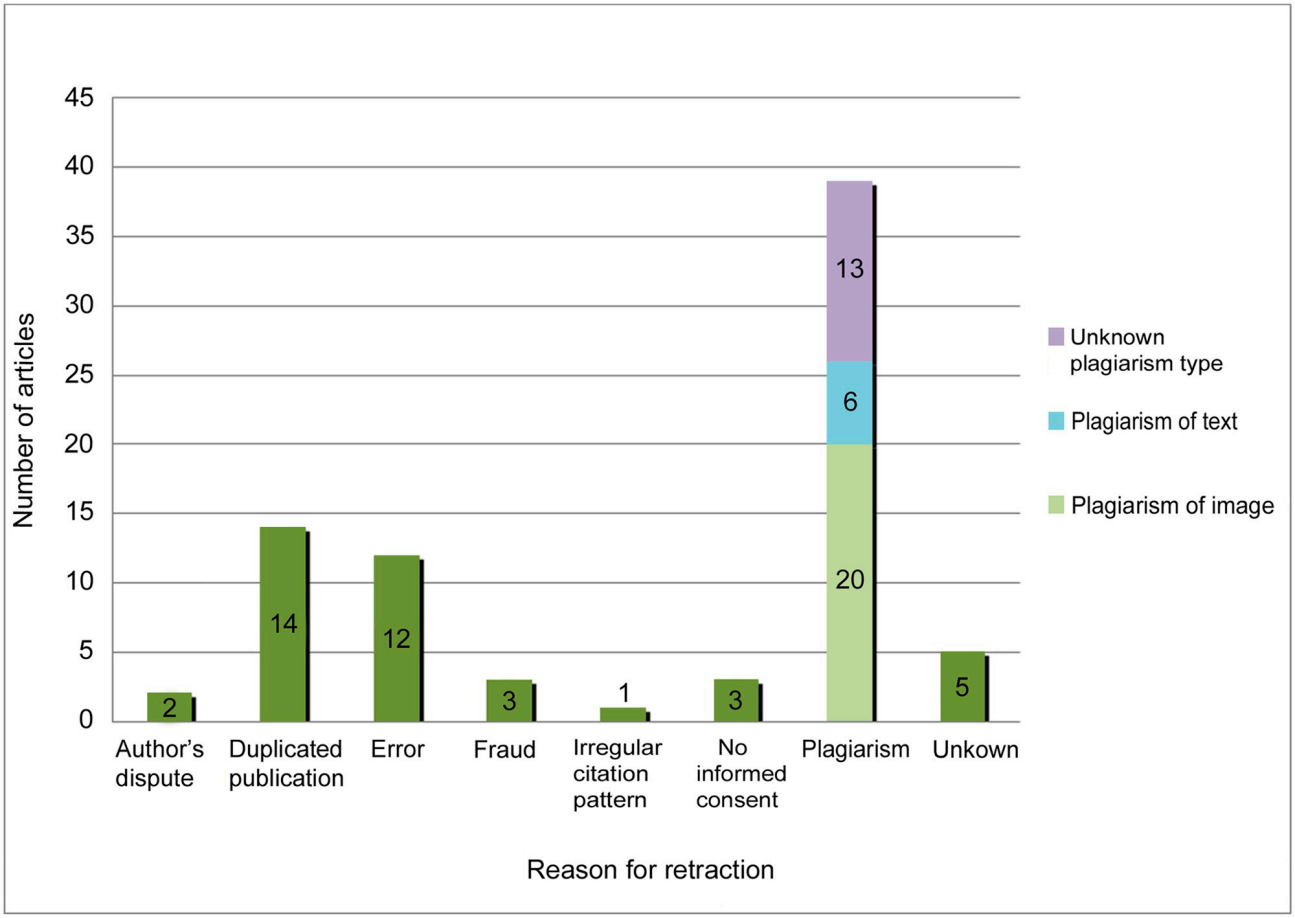
Count of articles by reason for retraction. Graphic representation: The distribution of number of articles by reason for retraction. Plagiarism is categorized under: a) unknown (purple bar), b) plagiarism of text (blue bar), c) plagiarism of image (light green bar).

The time to retraction varied from 0 to 19 years. Five retraction notices (7.7%), 3 from 2011 and 2 from 2012, did not specify the year of retraction. In 2017, one article was retracted less than a year after it was published (Fig 2).

The overall mean time to retraction was 3,36 years. Most articles (55%) took from one to three years from the time of publication to be retracted. Data showed the number of retracted articles increased significantly starting in 2012, the start point of this review.

### Number of citations after retraction

The analysis of post retraction citations is a proxy assessment of the influence of articles on scientific activity despite of their retraction. A total of 37% of the retrieved articles had a *positive-citation pattern*; meanwhile, 63% had a *negative-citation pattern*. The most cited article with a *negative-citation-pattern* was published in 2007 and was retracted in 2016 [24]. Thus far, it has received a total of 490 citations and of these, 58 were from after the retraction of the article.

### Association between impact factor and post retraction citation number

There was a strong positive correlation between the number of citations/year of an article after its retraction and the impact factor of the respective journal responsible for its retraction notice (Spearman rho = 0.69, p<0.05). The details of this analysis can be found in the S1 File.

### Association between the impact factor and the number of citations before the retraction

There was a moderate correlation between the number of citations/year of an article before its retraction and the impact factor of the journal in which it was published (Spearman rho = 0.43, p<0.05).

This review sample size did not allow for a multivariate analysis. The details of this analysis can be found in the S1 File.

### Quality of data from the retraction notices

Retraction notices are supposed to cite the original article [7]. However, our results showed that a proper citation of the original article was present in only 22 (33%) retraction notices; 42 retraction notices did not cite the original article; 1 article was cited three times in its retraction, implying that the retraction notice applied to more than one publication. Missing data were found in 57% of the retraction notices retrieved. Missing information in retraction notices was mainly regarding: date of retraction (7%), reason for retraction (7%), who requested the retraction (3%) and endorsement by the authors (38.4%). Retraction warnings such as a withdrawn/retracted red sign over the article were also nonexistent (37%).

### Reasons for retraction

The identified reasons for retraction are illustrated at Fig 2. Thirteen articles (20%) were retracted for at least two distinct reasons. Fraud was responsible for the retraction of three articles: two were retracted for image manipulation [16], [17] and one for data manipulation. Errors were attributed to inappropriate statistical analysis (n = 4), study design (n = 2) and inadequate data collection (n = 6). Retractions for duplicated publications were attributed to authors in 71% of the cases and to editors in 4,6% of the cases. Although an author’s dispute should not lead to a retraction [6], two articles accounted for retraction due to an author’s dispute. However, there is no additional information available for these retractions; therefore, it is not possible to assume this was the only reason for the retraction.

## Discussion

The comprehension of research integrity and the consequences of misconduct varies between different cultures [16], [17], [18]. Likewise, the concept of research integrity and research misconduct differ from institution to institution [2], [3]. In general, all institutions agree that fabrication, fraud and plagiarism negatively affect science to some extent, characterizing research misconduct [3], [13]; although, misconduct can have a wider definition [2]. Research integrity refers to a broader concept that does not necessarily imply misconduct or a direct effect on scientific integrity [13]. This diversity may explain the disparities between journals, publishers, research institutions, funders, and researchers when taking measures to prevent and report misconduct or breaches to research integrity. This scenario represents a challenge for academic studies on the matter.

In fact, for this review, the traditional bibliographic sources did not provide a complete picture of retracted articles. A total of eight (15%) articles were only identified on the Retraction Watch website, highlighting difficulties in retrieving retractions and suggesting poor transparency in the reporting of retractions.

Another obstacle of research transparency is the diversity of journal policies to deal with this subject [6], in that they do not always follow the COPE recommendation for the publication of retraction notices. For instance, the use of footnotes or comments from readers as an alert of a retraction [25], [26] and the absence of any type of warning in the database or in the article that is available in the journal. In addition, this review identified an erratum that was actually a retraction notice. These results reflect that some journal policies disregard research integrity flaws.

Legal threats to publishers have an influence on their positions regarding misconduct and, therefore, on the issue of retractions [7]. Despite publishers concern over litigation, this review found complete information, transparency and clarity of other retraction notices, supporting the existence of disparities between editors’ and publishers’ attitudes towards handling errors or misconduct.

The fact that public institutions funded the majority of the retracted articles also raises concerns regarding the importance of coordinated action between institutions to prevent research misconduct and to allocate a responsible investment of public funds.

### Reasons for retraction

In 2013, a Brazilian citation-stacking scheme used to increase journal impact factor was revealed [24]. Thompson and Reuters discovered that four journals were participating in self-citation in order to boost their impact factor [27]. Despite of the considerable number of retractions that were made as a result of this scheme, this review search strategy was able to identify a unique paper that was retracted for an irregular citation pattern [28], which is known as citation stacking. This fact addresses once more the difficulties in finding retracted articles [29], [30] and, therefore, warrants the necessity of efforts to maintain transparency in every step of scientific assembly.

Previous studies have shown that fraud and error have accounted for most of the retractions of biomedical articles [4], [28]; however, the present review revealed a larger number of retractions due to plagiarism. Fraud refers to fabrication, falsification or manipulation of data while error implies no intention to compromise the study [13]. Plagiarism may refer to unjust appropriation of ideas (text plagiarism) or images (image plagiarism). This review showed that 76% of the reported plagiarism was accounted for by image plagiarism. Among the cases of image plagiarism, 15% of the retractions clearly stated the existence of similarities of images to previous publications and raised manipulation concerns. In addition, 33.3% of the retractions due to plagiarism did not specify the type of plagiarism.

In regard to image editing, there is a fine line between what is allowed and what is not, and there are no standardized guidelines of scientific journals [13], [31]. Coordinated action is needed in order to establish guidelines and education for authors regarding image editing and the rationale for what is considered misconduct [32].

The underlying factors to explain why image plagiarism is the major cause of misconduct are unclear. Nevertheless, the notable increase in retractions is an indicator of the awareness of scientific misconduct [33] in regard to different forms of plagiarism and the necessity of actions to avoid this behavior.

### Are the increasing numbers of retracted publications a sign of scientific awareness of misconduct?

The results of this review are in accordance with those of previous studies about chronological trends of retracted publications [33], [34] that showed an increasing number of retractions in the past years. It is not possible to affirm that misconduct is increasing by evaluating only the retractions of authors affiliated with Brazilian institutions. Deeper investigation is needed to evaluate this aspect.

The increasing number of retracted publications over the years may be a sign of scientific awareness and response of authors, readers and institutions to flag questionable research [33], [34]. This can be illustrated by the request of authors to withdraw their article or the alert from other researchers to editors. In addition, more retractions are a reflex of advances in technology that can identify plagiarism and data manipulation [33], [34]. For instance, the use of software to identify image manipulation and plagiarism may increase the detection of such misconduct. Likewise, with a faster publication process, the publication of retractions and investigations–when needed–can be more efficient with the participation and collaboration of authors, institutions, researcher, and journals.

### What is the purpose of a retraction if not to be used to avoid more scientific misconduct?

A recent publication explored the nature of retracted articles [9]. The authors classified the citations as positive, neutral or negative. An interesting aspect of this study was the evaluation of a proper citation method for retracted articles. Otherwise, a retracted article is cited as legitimate and, hence, reliable. In most cases, it is not possible to assess whether a retracted article served as a basis for a new scientific investigation despite its retraction or whether it was cited without careful attention. Our finding regarding post retraction citation patterns showed how often retracted articles continue to receive positive citations without accurate retraction identification.

Further investigation is needed to understand why unreliable studies are still cited as legitimate [35]. Nevertheless, it is important to address that retracted publications might be used for new scientific production. A proper citation of retracted publications brings awareness to the causes involving its withdrawal and assists authors in not ignoring the retraction. Proper citation gives researchers the tools to make decisions in accordance with obvious ethical implications.

### The role of distinct actors in the publication of retractions

Retractions are published at the request of an author, publisher, editor, or community [4], [7], [8], [9]. The intention of a retraction is to promote transparency and clarity regarding research misconduct or an honest error that lead to flawed articles [4], [6], [7]. Thus, in accordance with the *COPE Guidelines for Retractions*, retractions should be published as soon as possible to avoid new citations of the unreliable work, researchers acting on its findings, or drawing more erroneous conclusions. Because the main goal is to minimize a chain of flaws, retractions should be transparent regarding the reason for the retraction, existence of endorsement by the authors, the date of retraction, a reference to the retracted article, a DOI, attachment to the original article and visibility [7], [36].

This review encompassed a wide range of retraction policies of different journals from the retraction wording to how the article is *red-flagged* [6], [7]. For wording, the reasons for the retraction were sometimes vague or absent. Information regarding retraction date and citation of the retracted article were also nonexistent for some publications. For methods to signal a retraction to readers, a variation from a big red note of *withdrawn/retracted* (*red-flag*) to a simple footnote was found. A possible explanation for the difficulties in retrieving articles for this review was the lack of a standardized publication of retraction notices. Furthermore, these practices are completely against the purpose of publishing retractions: transparency.

Endeavors to promote transparency are a caveat of unethical practices involving those involved in the scientific activity: scientists, publishers, editors, and academic institutions [18], [35], [36]; each has a specific role and may contribute to minimizing misconduct or not. Everybody has a role.

## Limitations and strengths

Incomplete information of the retraction notices reduced the accuracy of our analysis. Hence, the results obtained may underestimate the number of retractions due to restrictions of our search strategy, the level of transparency of the published retractions and their availability in the bibliographic databases.

Additionally, our analysis did not include an assessment of the original paper’s quality, and therefore, it is not possible to draw conclusions regarding the relationship between the research quality and retraction. Further investigations should be performed with this purpose since it is known that a retraction does not necessarily indicate a completely invalid study [1].

Since research integrity is a worldwide concern, despite the fact that this review considered only Brazilian institutions, its findings provide useful insights and could serve as a basis for future investigations.

## Conclusion

Retraction notices do not account only for research misconduct; they are also an alert of honest mistakes during scientific practices [6]. Nevertheless, these incidents compromise the quality and validity of research results. Considering authors affiliated with Brazilian institutions, this review concluded that most of the retractions of articles in health and life sciences were retracted for research misconduct.

Journals, funders, academic institutions, and researchers have an important educational and surveillance role to play in preventing research misconduct. The enforcement of disciplinary and educational measures is fundamental to reduce the incidence of corrupted science. In addition, the creation of a standard instrument for reporting retraction notices would assure the discussion of ethical policies and would promote a uniform publication of retractions.

This study attempted to emphasize the importance of coordinated action among all involved in scientific production in order promote research transparency. There is a positive impact of good practices when conducting investigations and reporting and publishing retraction notices. The underlying factors involving research misconduct remains unclear. Measures to prevent misconduct may take into consideration the particularities of each society, including weakness and strengths, depending on the cultural aspects. However, the impact of bad science is borderless and is not culture-dependent.

## Supporting information

S1 Table PRISMA checklist. (PDF)

S2 Table Study data. (XLS)

S1 File Statistical analysis pipelines and rationale. (PDF)
