# Supplementary material for: Research misconduct in health and life sciences research: A systematic review of retracted literature from Brazilian institutions
Source: PLoS One. 2019 Apr 15;14(4):e0214272. doi: 10.1371/journal.pone.0214272 (PMC6464327; doi:10.1371/journal.pone.0214272)
Supplement: S1 File — (PDF) [file pone.0214272.s003.pdf]

S1. Statistical analysis pipelines and rationale

All statistical analysis was performed using R programming version 3.4.2. The tests was conducted for the variables:

a) Number of citations BEFORE retraction

b) Number of citations AFTER retraction

c) Impact factor

1. Association between impact factor and number of citations before retraction

Pearson's correlation coefficient can be calculated for these two variables, however this coefficient can be used for population inference only when the joint distribution of variables is normal. Before conducting the test, assessment of the distribution and normality of each variable was performed. In case the variables did not behave normally, the use of a non-parametric test to evaluate correlation would be necessary (Spearman's correlation test).

1.1 Distribution of impact factor and number of citations before retraction

The graphic bellow suggests both variables have a linear positive correlation.

First data distribution was evaluated as followed:

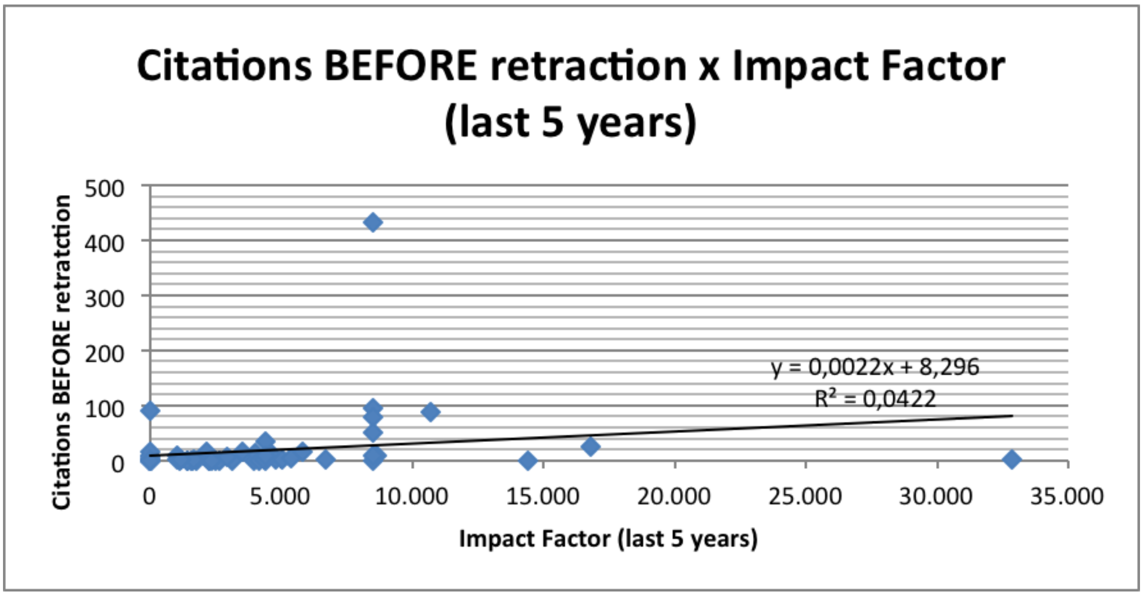

1.2 Assessment of normality for number of citations before retraction

To evaluate normality distribution of this variable, Shapiro-Wilk histogram was made. The test shows the variable is not normal. With a p-value < 0.05 the null hypothesis of normality is rejected.

The test result is bellow:

```
        Shapiro-Wilk normality test

data:  as.numeric(Dados$`Citations BEFORE retraction`)
W = 0.29479, p-value = 4.673e-16
```

1.3 Assessment of normality for impact factor

To evaluate normality distribution of this variable, Shapiro-Wilk histogram was made. The test shows the variable is not normal. With a p-value < 0.05 the null hypothesis of normality is rejected.

```
        Shapiro-Wilk normality test

data:  as.numeric(Dados$`Impact Factor (last 5 years)`)
W = 0.66832, p-value = 7.681e-11
```

1.4 Assessment of multivariate normality test

The multivariate normality test (for impact factor and number of citations before retractions) was performed using Henze-Zirkler test as follow:

```
           Test       HZ p value MVN
1 Henze-Zirkler 13.80451       0  NO
```

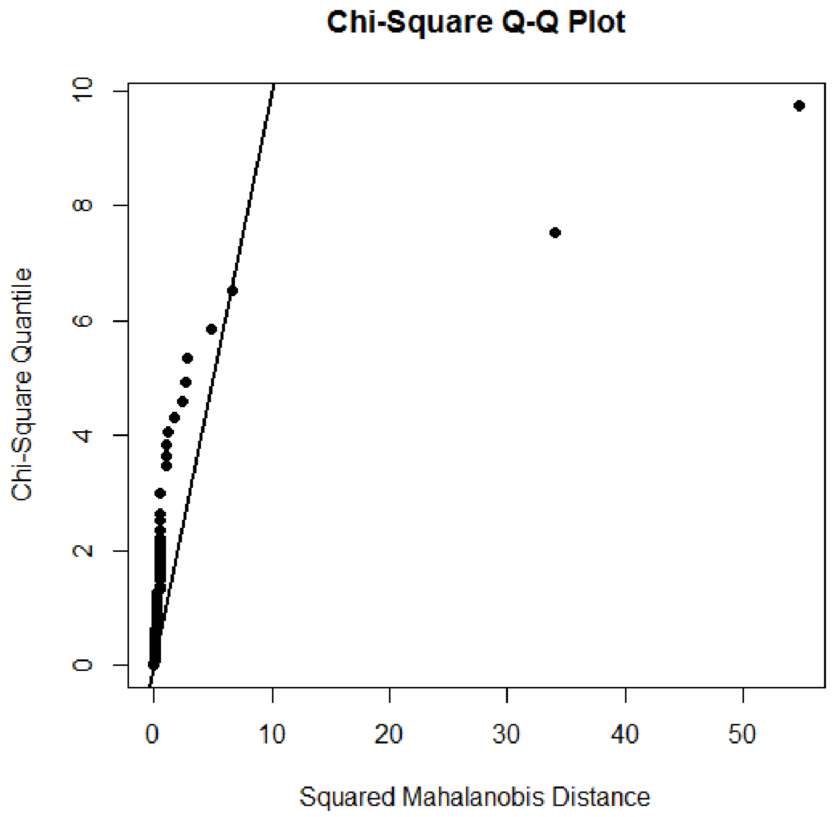

The test shows the variable is not normal. With p-value < 0,05 the null hypothesis of normality is rejected.

1.5 Use of Pearson's correlation test

Because the variables do not have a normal distribution, Pearson's correlation test can't be used for population inferences. However, it can still indicate the relation among the variables of this sample. Considering it, the test showed a correlation coefficient of 0.20. Therefore, the correlation is weak and positive. The test result is as follow:

```
                Pearson's product-moment correlation

data:  as.numeric(Dados$`Citations BEFORE retraction`) and
as.numeric(Dados$`Impact Factor (last 5 years)`)
t = 1.666, df = 63, p-value = 0.1007
alternative hypothesis: true correlation is not equal to 0
95 percent confidence interval:
 -0.04050932  0.42788096
sample estimates:
      cor
0.2054194
```

1.5 Use of Spearman's correlation test

Spearman's correlation test doesn't need a normal distribution of the variables involved. It is a non-parametric test that can be performed considering the variables distribution and the study sample size.

For it, the null hypothesis of no correlation between the variables considered $\rho = 0$. The result is as follow:

```
        Spearman's rank correlation rho


data:  as.numeric(Dados$`Citations BEFORE retraction`) and
as.numeric(Dados$`Impact Factor (last 5 years)`)

S = 26024, p-value = 0.0003346

alternative hypothesis: true rho is not equal to 0

sample estimates:

      rho

0.4313015
```

The test showed correlation coefficient of 0,43. It indicates a moderate correlation between impact factor and number of citations before retraction, p-value < 0.05.

2. Association between impact factor and number of citations after retraction

Pearson's correlation coefficient can be calculated for these two variables, however this coefficient can be used for population inference only when the joint distribution of variables is normal. Before conducting the test, assessment of the distribution and normality of each variable was performed. In case the variables did not behave normally, the use of a non-parametric test to evaluate correlation would be necessary (Spearman’s correlation test).

2.1 Distribution of impact factor and number of citations before retraction

The graphic bellow suggests both variables have a linear positive correlation.

First data distribution was evaluated as followed:

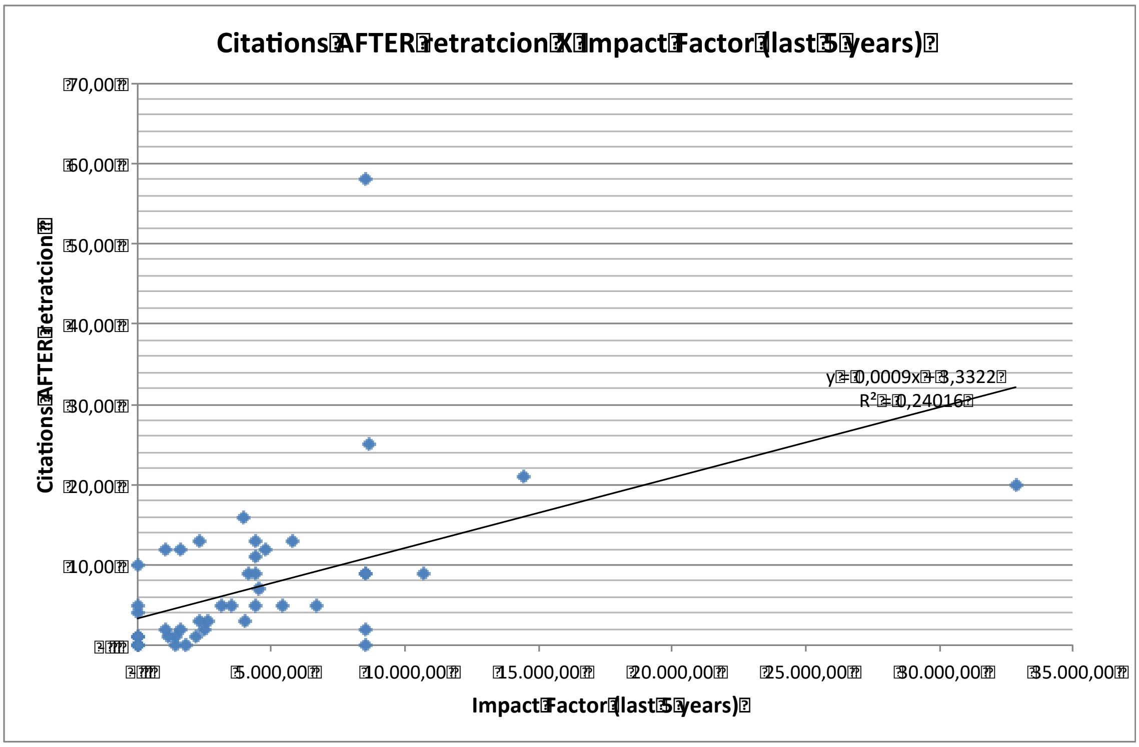

2.2 Assessment of normality for number of citations after retraction

To evaluate normality distribution of this variable, Shapiro-Wilk histogram was made. The test shows the variable is not normal. With p-value < 0.05 the null hypothesis of normality is rejected.

The test result is bellow:

```
Shapiro-Wilk normality test

data:  as.numeric(Dados$`N Citções pós retratação`)
W = 0.65856, p-value = 9.84e-10
```

2.3 Assessment of multivariate normality test

The multivariate normality test (for impact factor and number of citations after retractions) was performed using Mardia’s as follow:

```
Mardia's Multivariate Normality Test
```

```
--------------------------------------
   data : Dados2

   g1p             : 29.12677
   chi.skew        : 252.432
   p.value.skew    : 1.948166e-53

   g2p             : 43.5929
   z.kurtosis      : 32.08301
   p.value.kurt    : 0

   chi.small.skew : 277.4658
   p.value.small  : 7.84086e-59

   Result          : Data are not multivariate normal.
```

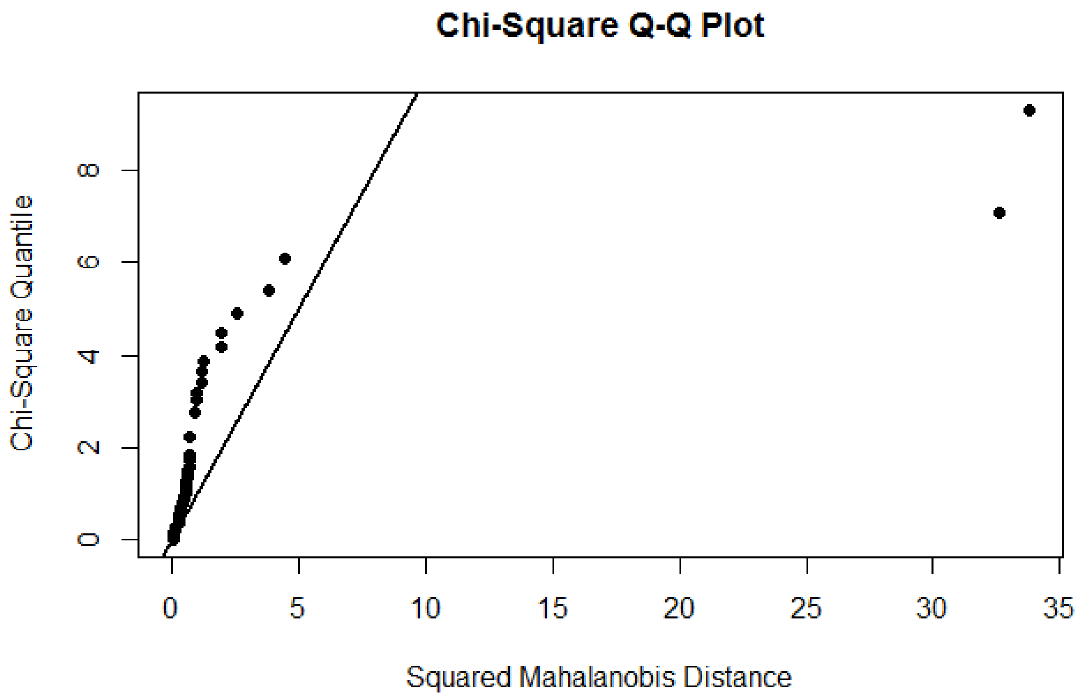

The test shows the variable is not normal. With a p-value < 0,05 the null hypothesis of normality is rejected.

2.4 Use of Pearson's correlation test

Because the variables do not have a normal distribution, Pearson's correlation test can't be used for population inferences. However, it can still indicate the relation among the variables of this sample. Considering it, the test showed a correlation coefficient of 0.56. It represents a moderate positive correlation. The test result is as follow:

```
Pearson's product-moment correlation

data:  as.numeric(Dados$`Citations AFTER retratcion`) and
as.numeric(Dados$`Impact Factor (last 5 years)`)
t = 5.3424, df = 63, p-value = 1.344e-06
```

```
alternative hypothesis: true correlation is not equal to 0
95 percent confidence interval:
 0.3640553 0.7061107
sample estimates:
      cor
0.5583748
```

2.5 Use of Spearman's correlation test

Spearman's correlation test doesn't need a normal distribution of the variables involved. It is a non-parametric test that can be performed considering the variables distribution and the study sample size. Hence, its results allow population inferences.

For it, the null hypothesis of no correlation between the variables considered $\rho = 0$. The result is as follow:

```
        Spearman's rank correlation rho

data:  as.numeric(Dados$`N Citções pós retratação`) and
as.numeric(Dados$`Fator de Impacto (ultimos 5 anos)`)
S = 6777.8, p-value = 3.587e-09
alternative hypothesis: true rho is not equal to 0
sample estimates:
      rho
0.7106711
```

The test showed correlation coefficient of 0.71. It indicates a strong correlation between impact factor and number of citations after retraction, p-value < 0.05.
